# Expression of estrus as a relevant factor in fixed-time embryo transfer programs using estradiol/progesterone-based protocols in cattle

**DOI:** 10.21451/1984-3143-AR2018-0060

**Published:** 2018-08-16

**Authors:** Gabriel A. Bó, Andrés Cedeño

**Affiliations:** 1 Instituto de Reproducción Animal Córdoba (IRAC), Zona Rural General Paz, Córdoba, Argentina.; 2 Instituto de Ciencias Básicas, Medicina Veterinaria, Universidad Nacional de Villa María, Villa del Rosario, Córdoba,Argentina.; 3 Maestría en Reproducción Bovina, Facultad de Ciencias Agropecuarias, Universidad Nacional de Córdoba, Argentina.; 4 Medicina Veterinaria, Escuela Superior Politécnica Agropecuaria de Manabí (ESPAM), Calceta, Manabí, Ecuador.

**Keywords:** embryo-fetal losses, estrus, recipients

## Abstract

The main objective of implementing embryo transfer in beef operations is to accelerate the rate of genetic progress in the herd. Among the main factors that affect the use of these technologies are related to nutrition, management and estrus synchronization. As a result of research conducted over the last 20 years, recipient utilization has increased by applying protocols that synchronize ovulation and allow for embryo transfer without the need for estrus detection, usually referred to as fixed-time embryo transfer (FTET). Although these protocols have performed adequately for several years, recent attention has been directed to the effect of estrus expression and estradiol concentrations during growth of the preovulatory follicle on embryo growth and pregnancy. The experiments reviewed herein demonstrate that estrus expression is associated positively with high pregnancy rates and reduced pregnancy losses in recipients receiving *in vitro*-produced and *in vivo* -derived frozen/thawed bovine embryos.

## Introduction

The main objective of an embryo transfer program is to increase the genetic value of the offspring produced in a given herd. Nutrition, management and efficiency in the detection of estrus are among the factors that affect the use of these technologies ( Mapletoft and Bó, 2016 ). The protocols that synchronize estrus and ovulation have allowed for embryo transfer at predetermined time, without the requirement for estrus detection. These protocols are usually referred to as fixed-time embryo transfer (FTET; Bó *et al*., 2002 , 2012a ). Although efforts to synchronize ovulation have resulted in cows ovulating in a shorter time interval as compared to untreated cycling animals, ovulation without expression of estrus often occurs. The objective of this manuscript is to briefly review protocols that are used to synchronize ovulation, and discuss how the expression of estrus may impact on pregnancy in embryo recipients.

## Conventional synchronization treatments for embryo recipients in South America

Although prostaglandin F2α (PGF2α) has been used most commonly for synchronization of estrus, the requirement for estrus detection and the variability in the interval from treatment to estrus and ovulation has adversely affected its performance in embryo transfer programs, especially in *Bos indicus* cattle (reviewed in Bó *et al*., 2002 ).

To avoid limitations associated with estrus detection, treatments that synchronize the time of ovulation, which were developed originally for fixed-time AI, have been utilized for FTET. These treatments are generally divided into those that are GnRH-based ( Ambrose *et al*., 1999 ) and those that are estradiol-based ( Bó *et al*., 2002 ). In either case, the recipient protocols include the insertion of a progesterone (P4) releasing device for 7 or 8 days ( Hinshaw, 1999 ; Bó *et al*., 2002 ).

Estradiol and P4 (estradiol/P4)-based treatments are the most commonly used protocols to synchronize follicle wave emergence and ovulation of recipients in South America ( Baruselli *et al*., 2010 ). The simplified protocol used most commonly nowadays consists of insertion of a P4-releasing device and the administration of 2 mg estradiol benzoate (EB) on day 0, and PGF2α at the time of insertion and removal of the P4-device if it is impregnated with >1 g of P4 and only at P4 device removal when it contains <1 g of P4. The P4-device is usually removed on day 7 or 8 and 300 or 400 IU of equine Chorionic Gonadotropin (eCG) is given at that time ( Bó *et al*., 2002 ). Ovulation is induced by the administration of 0.5 or 1 mg of estradiol cypionate (ECP) at the time of P4-device removal and all recipients with a CL receive an embryo 9 days later (i.e., 7 days after the expected time of estrus; Baruselli *et al*. 2010 , 2011 ; Bó *et al* 2012a , b ).

Overall, 75 to 85% of the recipients treated with this protocol receive an embryo (compared to 50% or less with PGF2α synchronization), P4 concentrations are high at the time of embryo transfer and pregnancy per embryo transfer (P/ET) usually exceeds 50%, when both embryos and recipients are of high quality (reviewed in Bó *et al*., 2002 ; Baruselli *et al*., 2010 , 2011 ).

## Protocols that prolong the proestrus period

Recent studies have suggested that reducing the length of exposure of the P4-releasing device insertion to 5 days and increasing the interval from P4-device removal to GnRH and fixed-timed AI to 3 days may improve pregnancy per AI (P/AI) as compared to the traditional 7-day GnRH/P4 device protocol in beef cattle ( Bridges *et al*., 2008 ). Furthermore, it was suggested that a reduction in the length of the growth phase of the ovulatory follicle prior to ovulation, as occurs in some animals treated with the conventional 7-day protocols, alters the steroidogenic capacity of the dominant follicle prior to ovulation and the resulting CL, and decreases the ability of the uterus to support embryo development (reviewed in Bridges *et al*., 2013 ). Using a modified 5-day Co-Synch+CIDR protocol (no GnRH at P4 device insertion, PGF2α at P4 removal on day 5 and GnRH on day 8) Sala *et al*. (2016) reported similar P/ET rates with *in vitro*-produced embryos to those of recipients synchronized with two PGF2α treatments 14 days apart and estrus detection. Based on these findings, we evaluated the effectiveness of an estradiol/P4 treatment protocol in which the exposure to P4 device was reduced to 6 days and proestrus was lengthened by the administration of GnRH 72 h after P4 device removal instead of ECP at device removal. The protocol for FTAI was named J-Synch ( de la Mata and Bó, 2012 ). This treatment protocol has resulted in higher P/AI rates in beef heifers compared to the conventional protocol in which the P4-device is removed on day 7 and ECP is given at that time ( Bó *et al*., 2016 ).

A series of studies have been conducted recently to evaluate the performance of the J-Synch protocol in embryo transfer programs ( Menchaca *et al*., 2015 , 2016 ). The experiments were performed in Uruguay with 3,782 cycling *Bos taurus* beef recipients that received by FTET Holstein embryos produced *in-vitro* with sex-sorted semen. The first experiment compared pregnancy rates obtained with J-Synch protocol to the conventional estradiol/P4 protocol for FTET. All recipients received a P4-device (0.5 g progesterone, DIB 0.5, Zoetis, Uruguay) plus 2 mg EB (Gonadiol, Zoetis) i.m on day 0. In the J-Synch group (n = 464), the DIB was removed on day 6 and 500 µg cloprostenol (PGF2α, Ciclase DL, Zoetis) and 400 IU eCG (Novormon, Zoetis) were given i.m and GnRH analogue (100 µg gonadorelin acetate, Gonasyn GDR, Zoetis) was given 72 h later. In the conventional treatment (n = 481), the DIB was removed on day 7 and PGF2α, eCG, and 0.5 mg ECP (Cipiosyn, Zoetis) were administrated i.m. at the same time. The second experiment compared GnRH *vs* . EB to induce ovulation in the J-Synch protocol. The J-Synch protocol was performed as described previously with GnRH given at 72 h (n = 456) or 1 mg EB given at 60 h after P4 device removal (n = 461). In experiment 3, we evaluated the effect of the time of GnRH administration in the J-Synch protocol (GnRH was given at 60 h (n = 452) or 72 h (n = 466) after device removal). For all the experiments, recipients received *in vitro*-produced embryos on day 16-17 by FTET, and P/ET was determined by ultrasonography 40-50 days later. Overall, the P/ET rate was higher in the J-Synch (49.4%, 229/464) than in the conventional synchronization protocol (41.0%, 197/481; P < 0.05) group regardless of whether GnRH was administered at 72 h (58.8%, 230/391) or EB was administered at 60 h (54.7%, 227/415); or whether GnRH was administered at 60 h (47.8%, 216/452) or 72 h (50.4%, 235/466). However, results suggest that prolonging the exposure to endogenous estradiol prior to ovulation, as it occurs with the J-Synch protocol, improved P/ET with *in vitro*-produced embryos. It was also noteworthy that in a fourth study, in which cows (n = 581) were treated with the J-Synch protocol (as described previously), but with some recipients being allowed to ovulate spontaneously (n = 532), P/ET was greater for recipients that did not receive GnRH (57.5%, 306/532 *vs*. 51.5%, 299/58; P < 0.05). Results suggest that shortening the growth period of the ovulatory follicles with GnRH may adversely affect the chances of pregnancy in some cows. Thus, waiting for the natural expression of estrus may improve P/ET.

## Expression of estrus, preovulatory estradiol and the establishment of pregnancy

Estrus is defined as the period in which a female is sexually receptive and it is due to increased circulating concentrations of estradiol at a time when P4 concentrations are low ( Allrich, 1994 ). Furthermore, it has been suggested that the progression of events required for conceptus growth, elongation, survival and attachment are influenced by the coordination of events leading to a decrease in P4 concentrations and an increase in estradiol concentrations prior to the onset of estrus ( Bridges *et al*., 2013 ). Additionally, preovulatory estradiol concentrations have been reported to have a positive impact on subsequent conceptus development, and cows that exhibit estrus have been reported to have a greater conceptus length on day 19 of gestation compared to those not exhibiting estrus ( Davoodi *et al*., 2016 ).

It has been shown previously that the occurrence of estrus in FTAI programs is positively associated with P/AI in *Bos taurus* ( Richardson *et al*., 2016 ) and *Bos taurus x Bos indicus* beef cattle ( Bó *et al*., 2017 ), and with the diameter of the dominant follicle at FTAI, the diameter of the subsequent CL, the P4 concentrations in the luteal phase and P/AI in *Bos indicus* beef cattle ( Sá Filho *et al*., 2011 ). Furthermore, diameter of the dominant follicle at the time of FTAI has been associated with pregnancy success in both *Bos taurus* ( Lamb *et al*., 2001 ; Perry *et al*., 2004 , 2005 , 2007 ) and *Bos indicus* (Sa Filho *et al*., 2010). However, when postpartum beef cows were inseminated based on standing estrus, ovulatory follicle size had no influence on pregnancy success ( Perry *et al*., 2005 ), indicating again that high estradiol concentrations produced by the dominant ovulatory follicle are essential for high P/AI ( Perry *et al*., 2014 ). More specifically, follicle maturity may affect fertility through the preparation of the oocyte for embryo development, preparation of follicular cells for luteinization, and/or preparation of the uterine environment for the establishment of pregnancy. Therefore, the expression of estrus may affect P/ET in recipients synchronized with FTET protocols.

It has been previously shown that expression of estrus in *Bos indicus x Bos taurus* recipients treated with the Ovsynch protocol resulted in greater diameter of the ovulatory follicle, subsequent CL area and P4 concentrations and greater P/ET than in those that did not show estrus ( Baruselli *et al*., 2003 ). The reasons for the higher P/ET in the recipients showing estrus is that they were exposed to higher estradiol concentrations than those that were induced to ovulate with GnRH prior to showing estrus and the higher P4 concentrations at the time of FTET. In a recent study involving the reciprocal embryo transfer between donor cows and recipients induced to ovulate either a large or small dominant follicle with GnRH revealed interesting results ( Atkins *et al*., 2013 ). In the study of Atkins *et al*. (2013) , single embryos (n = 354) that were obtained from cows induced to ovulate a large or a small follicle with GnRH were transferred into recipients that were also induced to ovulate a small or a large follicle with GnRH. Pregnancy maintenance from 7 to 27 days of gestation was enhanced by increased serum estradiol concentrations at the time of the GnRH treatment and P4 concentration 7 days later in the recipient cows. However, this study also showed that follicle diameter was not all that important, as recipients with large follicles had the lowest pregnancy rates, indicating that estradiol produced by a new growing dominant follicle will benefit pregnancy more than an aged large dominant follicle that has already reduced estradiol production at the time of GnRH-induced ovulation ( Bridges *et al*., 2014 ). In another study, donor and recipient cows were retrospectively divided based on their plasma estradiol concentrations at induced ovulation ( Jinks *et al*., 2013 ). In this study, circulating estradiol concentrations of the recipient cows, not the donor, was related to higher pregnancy rates, indicating that the primary benefit of increased preovulatory estradiol concentrations is mediated through alterations in the uterine environment of the recipient cows. Finally, two other studies in which estradiol treatments were administered to increase circulating estradiol concentrations prior to ovulation were conducted. Jinks *et al*. (2013) reported that the administration of 0.5 mg ECP 24 h before AI increased P/AI in cows induced to ovulate a small dominant follicle (<12.2 mm) with GnRH. Madsen *et al*. (2015) treated ovariectomized cows with CIDR devices (1.38 g of P4, Zoetis, USA) to mimic the luteal phase and then treated with 2.5 mg ECP 12 h after CIDR removal, 1.2 mg EB 36 h after CIDR removal or no treatment (CON) to mimic a preovulatory period. Ovulation was simulated with an injection of GnRH 48 h after CIDR removal and embryos were transferred on 7 days later. Pregnancy was maintained by administration of injectable P4 from days 3 to 6 and then CIDR devices until day 29. Cows that received estradiol treatments had greater embryonic survival and P/ET compared to control animals (4, 29, and 21% for CON, EB, and ECP, respectively; Madsen *et al*., 2015 ).

## Expression of estrus and pregnancy rates in recipients treated with estradiol/P4-based FTET protocols

After reviewing the previous studies, the effect(s) of estradiol concentrations and estrus expression on pregnancy rates in recipients in South America is unclear, since most are treated with estradiol/P4-based protocols. Furthermore, it would seem to be important to differentiate whether cows are induced to ovulate with EB or ECP; the resulting plasma estradiol-17β concentrations would be much higher for a shorter period of time in cows treated with EB as opposed to ECP (reviewed in Bó *et al*., 2013 ). In that regard previous results from our lab ( Bó *et al*., 2012b ) and others ( Looney *et al*., 2010 ) have shown no significant effects of expression of estrus on P/ET in recipients receiving EB as compared to the shorter acting estradiol-17β to induce ovulation.

A retrospective analysis of several experiments performed on 13 different commercial dairy farms in Brazil using an estradiol/P4-based protocol but with ECP rather than EB to induce ovulation revealed a positive association between estrus expression and fertility ( Pereira *et al*., 2016 ). Pereira *et al*. (2016) observed that lactating dairy cows were either artificially inseminated (n = 5430) or used as recipients (n = 2003). All cows were treated with a CIDR (Zoetis, Brazil) and 2 mg EB on day 0, 25 mg of dinoprost (Lutalyse, Zoetis) on day 7, and CIDR removal and 1 mg of ECP (Zoetis) on day 9. Cows were either FTAI on day 11 or FTET with fresh *in vitro*-produced embryos on day 18. Estrus was detected using tail-head devices (Estrotec, Rockway Inc., Spring Valley, WI) and pregnancy was determined on days 32 and 60 of gestation. Estrus expression positively influenced (P < 0.01) P/AI on day 32 of gestation (estrus 38.9%, 1785/4584 *vs* . no estrus 25.5%, 222/846) and P/ET (estrus 46.2%, 645/1397 *vs*. no estrus 32.7%, 193/606). Furthermore, pregnancy loss to day 60 was lower (P < 0.01) in cows that expressed estrus in FTAI (estrus 14.4%, 255/1785 *vs*. no estrus 20.1%, 43/222) and FTET (estrus 18.6%, 120/645 *vs*. no estrus 22.7%, 43/193). Similar results were also reported with crossbred *Bos indicus x Bos taurus* beef heifers; the manifestation of estrus behavior up to 3 days after P4 device removal increased the probability of pregnancy in recipients receiving IVP embryos ( Frade *et al*., 2014 ). Heifers expressing standing estrus had greater P/ET than heifers that did not express estrus (62.4%, 106/170 *vs*. 47.0%, 31/66; P ≤ 0.01). In addition, heifers that became pregnant had greater circulating P4 concentrations at FTET (2.8 ± 0.14 ng/ml; n = 137) than those that did not become pregnant (2.2 ± 0.18 ng/ml; n = 99; P = 0.04; Frade *et al*., 2014 ). Thus, the sequential exposure to greater concentrations of estradiol during the pre-ovulatory phase and the subsequent exposure to high P4 in the diestrus positively influenced pregnancy success after FTET.

The potential practical drawback of these results was that doing embryo transfer in only the recipients that express estrus would reduce the proportion of recipients transferred/treated, which has been shown as one of the main benefits of using FTET programs in recipients in South America ( Bó *et al*, 2002 , 2012b ; Baruselli *et al*., 2010 , 2011 ).

Two other studies were performed in Argentina to confirm that the expression of estrus had a positive effect on P/FTET and maintenance of pregnancy in recipients treated with estradiol/P4-based protocols. A secondary objective was to evaluate if the administration of GnRH at the expected time of estrus to recipients not showing estrus would increase the proportion of recipients transferred and pregnant. In the first experiment, 729 non-lactating beef cows (Bonsmara x *Bos indicus*, Brangus and Braford) received a DIB 0.5 g device (Zoetis, Argentina) plus 2 mg of EB on day 0 and on day 8, DIBs were removed and 400 IU of eCG (Novormon 5000, Zoetis) plus 0.5 mg of ECP (Cipiosyn, Zoetis) and 500 μg of cloprostenol (Ciclase DL, Zoetis) were administered ( Cedeño *et al*. 2017 ). All cows were tail-painted (CeloTest, Biotay S.A., Argentina) for estrus detection (>30.0% paint loss = estrus). All recipients not showing estrus by 48 h (paint loss ≤30.0%) were randomly divided to receive GnRH (100 μg of gonadorelin acetate; Gonasyn GDR, Zoetis) or no treatment. Estrus was again detected 56 h after P4 device removal and was recorded. On day 17, cows were examined by ultrasonography and those with a CL ≥18 mm (grade 1), ≥ 16 and <18 mm (grade 2) and ≥14 and <16 mm in diameter (grade 3) received *in vivo*-derived, frozen/thawed or *in vitro*-produced fresh embryos by non-surgical transfer. The overall proportion of recipients transferred was 88.1% (583/729) and the overall P/ET 46.0% (268/583). The proportion of recipients in estrus at 48 and 56 h after P4-device removal was 87.6% and P/ET was higher (P<0.05) in recipients showing estrus (48.3%, 250/518) than in those not showing estrus (30.1%; 22/73; Table 1 ). When CL diameter at the time of FTET was considered, P/ET did not differ in recipients showing estrus that had a CL ≥18 mm or between 16 and 18 mm; however, P/ET was lower in those not showing estrus, even in those with a CL ≥18 mm in diameter at the time of FTET (P < 0.05, Table 1 ).

**Table 1:** Effect of expression of estrus and CL diameter at the time of FTET on P/ET in recipient beef cows treated with an estradiol/P4-based synchronization protocol ^*^ .

	CL diameter (mm)			
	≥18	≥16 & <18	≥14 & <16	Total
Estrus	50.0%a (203/406)	44.2%ab (46/104)	13.0%c (1/8)	48.3%a (250/518)
No estrus	37.0%b (15/41)	24.1%b (7/29)	0%d (0/3)	30.1%b 22/73
Total	48.8%a (218/447)	40.0%a (53/133)	9.1%b (1/11)	

abcdDenotes significant differences within rows and columns (P < 0.05).

* All cows received 400 IU eCG, 500 µg cloprostenol and 0.5 mg ECP at P4 device removal and observed for estrus (i.e. tail- paint loss) 48-56 h later.

When the GnRH treatment was considered in recipients that did not expressed estrus by 48 h after DIB removal, P/ET was significantly higher (P < 0.05) in those treated with GnRH (34/74, 46.0%) than in those not treated with GnRH (12/46, 26.0%). However, when the expression of estrus at 56 h after DIB removal was considered, P/ET were higher in those showing estrus, whether or not they received GnRH (26/48, 54.2%) than in those not showing estrus (15/43, 34.9%); and although the numbers were low, GnRH treatment did not appear to increase P/ET in recipients that did not show estrus (GnRH: 38.0%, 8/21 *vs*. no GnRH: 31,8%, 7/22).

A second study was conducted using non-lactating crossbred beef cows (205 Brangus and 198 Braford; Cedeño *et al*., 2018 ). On day 0 all animals received a DIB 0.5 device plus 2 mg EB and then recipients were randomly allocated into two treatment groups. In recipients treated with the conventional estradiol/P4 synchronization protocol P4 devices were removed on day 8 and also received PGF2α, eCG and ECP at the same time. In recipients treated with the J-Synch protocol P4 devices were removed on day 6 and received PGF2α and eCG at the same time. All recipients were tail-painted and those not showing estrus by 48 h (conventional group) or 62 h (J-synch group) received GnRH at that time. Recipients were again run through the chute for tail-paint observation and recording 8 h later in both treatment groups. All cows with a CL received an *in vitro-*produced embryo by FTET 7 days after an observed estrus or GnRH treatment. The overall proportion of recipients transferred was 86.5% (352/407) and the overall P/ET was 37.8% (133/352). Although the proportion of recipients transferred was higher (P < 0.05) in the conventional group 90.0% (180/201) than in the J-Synch group 83.5% (172/206), no significant differences were found in P/ET between the two synchronization protocols (Conventional: 36.6%, 66/180 *vs*. J-Synch 39.0%, 67/172). However, the most important finding of this experiment was the great difference in P/ET and pregnancy losses between the recipients that showed estrus, regardless of treatment group. Recipients showing estrus had significantly higher (P < 0.05) P/ET than those that did not show estrus ( Table 2 ). Furthermore, recipients not showing estrus had a higher rate of embryonic/fetal losses between 30 and 60 days (P = 0.004) of gestation and consequently a lower P/ET at 60 days and a lower calving rate (P < 0.01; Table 3 ).

**Table 2:** Pregnancy/ET as a function of the manifestation of estrus in recipients treated with two estradiol-based synchronization protocols.

	Synchronization Treatment		
	Conventional ^*^	J-Synch ^**^	Total
Estrus	38,3% (62/162)	40,0% (62/155)	39,1%a (124/317)
No Estrus	22,2% (4/18)	29,4% (5/17)	25,7%b (9/35)

abMeans within columns with different superscripts differed (P < 0.05).

* Recipients in the Conventional protocol received 0.5 mg ECP at P4 device removal (Day 8) and were observed for estrus (i.e. tail-paint loss) 48 and 56 h later.

** Recipients in the J-Synch protocol did not receive ECP at P4 device removal (Day 6) and were observed for estrus 62 and 70 h later. All recipients not showing estrus by 48 h (Conventional) or 62 h (J-Synch) received GnRH at that time.

**Table 3:** Effect of estrus expression on P/ET at 30 and 60 days of gestation, 30 to 60-d and 60-calving pregnancy loses and calving rate in recipients synchronized with two estradiol/P4-based treatments.

	P/ET (30 days)	P/ET (60 days)	Pregnancy loss (30 to 60 days)	Pregnancy loss (60 days to calving)	Calving rate
Estrus	39.1%a (124/317)	37,0%a (117/317)	5,6%a (7/124)	20,5%c (24/117)	29,3%a (93/317)
No Estrus	25,7%b (9/35)	8,6%^b^ (3/35)	66,7%b (6/9)	66,7%d (2/3)	2,9%b (1/35)
Total	38,0% (133/35)	34,1% (120/352)	9,8% (13/133)	21,7% (26/120)	26,7% (94/352)

abDenotes significant differences within columns (P < 0 .01). ^cd^Denotes a tendency to differ (P < 0.06).

Results did not confirm previous studies in which P/ET rates were 8.5% with the J-Synch protocol ( Menchaca *et al*., 2015 ). In fact, the small difference in favor of the J-Synch group was overridden by the higher proportion of recipients that were transferred in the conventional group. The differences in the proportion of recipients transferred/treated could be due to a higher ovulation rate in cows with small follicles that received ECP rather than GnRH ( Jinks *et al*., 2013 ). However, the most interesting finding in the four studies reviewed in this section was the difference in P/ET between the recipients showing estrus and those not showing estrus, suggesting that the expression of estrus in FTET programs is linked to a uterine environment that favors embryo development (reviewed by Bridges *et al*., 2013 and Perry, 2017 ). As stated previously, even though recipients received ECP at the time of P4 device removal, those that manifested estrus probably had higher plasma estradiol concentrations resulting from ECP administration and from the developing ovulatory follicle than those not showing estrus. Therefore, recipients that showed estrus had sufficient estradiol exposure for the required changes in uterine environment for embryonic growth, survival and the establishment of pregnancy ( Bridges *et al*., 2013 ).

This last study and that from Pereira *et al*. (2016) both showed a significantly higher rate of pregnancy loss in recipients not showing estrus.

Surprisingly, pregnancy losses after 60 days were also higher in recipients not showing estrus. Embryo/fetal losses as high as 17.2% from day 32 to day 60 of pregnancy have been reported in dairy heifers (Garcia Guerra *et al*., 2016) and between 0 to 34,5% in beef cows ( Tribulo et al., 2017 ) carrying IVF embryos. However, none of these reports related estrus expression to pregnancy losses from 60 days of gestation to term. During this period there is a continuous growth of the fetus and also dramatic changes in the placenta to meet the increasing nutritional demands of the growing fetus (reviewed by Wiltbank *et al*., 2017 ). Estimates of pregnancy loss during this period could range from 3-5% in recipients carrying *in vivo*-derived embryos to as much as 20% in recipients carrying *in vitro*-produced embryos (Bó and Cedeño, 2018, IRAC, Córdoba, Argentina, unpublished data). Factors associated with increased pregnancy loss in this period have not been studied in detail; however, the possibility of several infectious agents that can affect pregnancy during this period of time cannot be ruled out. Twinning was identified in one study as a major contributor to the pregnancy loss during this period (Lopez Gatius *et al*., 2004). However, this is not the case in the presently described study because all recipients received single embryos. Although other reasons may be related to the *in vitro*-produced embryo itself ( Miles *et al*., 2005 ), this would not explain the differences observed between the recipients that did or did not show estrus. Obviously, more studies are required to confirm these findings.

## Summary and final conclusions

The protocols developed for FTET over the last 20 years have provided practitioners the greatest opportunity for transferring large number of embryos in recipient herds and has been pivotal for the development of the large scale IVF embryo-derived industry in South-America. Although overall pregnancy rates have been considered adequate for most practitioners, there are factors such as the expression of estrus that need to be considered for successful embryo transfer programs, especially in protocols in which ECP is used to induce ovulation or no estradiol is administered at the time of P4-device removal. The present manuscript has reviewed several studies showing a positive correlation between the manifestation of estrus, pregnancy rates and pregnancy maintenance in recipients. The use of tail-paint or estrus detection patches in recipients would help identify animals showing estrus by simply running them through the chute at the appropriate time, without the necessity of labor intensive estrus observations. These modifications can be easily implemented in recipient synchronization programs and should result in overall higher pregnancy rates.

## References

[B001] Allrich RD (1994). Endocrine and neural control of estrus in dairy cows. *J Dairy Sci*.

[B002] Ambrose JD, Drost RL, Monson RL, Rutledge JJ, Leibfried-Rutledge ML, Thatcher MJ, Kassa T, Binelli M, Hansen PJ, Chenoweth PJ, Thatcher WW (1999). Efficacy of timed embryo transfer with fresh and frozen in vitro-produced embryos to increase
pregnancy rates in heat-stressed dairy cattle. *J Dairy Sci*.

[B003] Atkins JA, Smith MF, MacNeil MD, Jinks EM, Abreu FM, Alexander LJ, Geary TW (2013). Pregnancy establishment and maintenance in cattle. *J Anim Sci*.

[B004] Baruselli PS, Marques MO, Carvalho NAT, Berber RCA, Valentim R, Carvalho Filho AF, Costa Neto WP (2003). Dinâmica folicular e taxa de prenhez em novilhas receptoras de embrião
(*Bos taurus indicus* x *Bos taurus taurus*) tratadas
com o protocolo Ovsynch para inovulação em tempo fixo. *Braz J Vet Res Anim Sci*.

[B005] Baruselli PS, Ferreira RM, Sá Filho MF, Nasser LFT, Rodrigues C, Bó GA. (2010). Bovine embryo transfer recipient synchronisation and management in tropical environments. Reprod Fertil Dev.

[B006] Baruselli PS, Ferreira RM, Sales JNS, Gimenes LU, Sá Filho MF, Martins CM, Rodrigues CA, Bó GA. (2011). Timed embryo transfer programs for management of donor and recipient cattle. Theriogenology.

[B007] Bó GA, Baruselli PS, Moreno D, Cutaia L, Caccia M, Tríbulo R, Tríbulo H, Mapletoft RJ (2002). The control of follicular wave development for self-appointed embryo transfer programs
in cattle. Theriogenology.

[B008] Bó GA, Baruselli PS, Mapletoft RJ (2012). Increasing pregnancies following synchronization of bovine recipients. Anim Reprod.

[B009] Bó GA, Peres LC, Cutaia LE, Pincinato D, Baruselli PS, Mapletoft RJ (2012). Treatments for the synchronisation of bovine recipients for fixed-time embryo transfer
and improvement of pregnancy rates. Reprod Fertil Dev.

[B010] Bó GA, Baruselli PS, Mapletoft RJ (2013). Synchronization techniques to increase the utilization of artificial insemination in
beef and dairy cattle. Anim Reprod.

[B011] Bó GA, de la Mata JJ, Baruselli PS, Menchaca A (2016). Alternative programs for synchronizing and re-synchronizing ovulation in beef cattle. Theriogenology.

[B012] Bó GA, Cedeño A, Tribulo A, Andrada S, Tribulo R, Mapletoft RJ (2017). Influence of estrus expression and treatment with GnRH on pregnancy rates in beef cattle
synchronized with progesterone devices and estradiol and inseminated at a fixed time. Reprod Fertil Dev.

[B013] Bridges GA, Helser LA, Grum DE, Mussard ML, Gasser CL, Day ML (2008). Decreasing the interval between GnRH and PGF2α from 7 to 5 days and lengthening proestrus
increases timed-AI pregnancy rates in beef cows. *Theriogenology*.

[B014] Bridges GA, Day ML, Geary TW, Cruppe LH (2013). Deficiencies in the uterine environment and failure to support embryonic development. *J Anim Sci*.

[B015] Bridges GA, Mussard ML, Hesler LA, Day ML (2014). Comparison of follicular dynamics and hormone concentrations between the 7-day and 5-day
CO-Synch + CIDR program in primiparous beef cows. *Theriogenology*.

[B016] Cedeño A, Tribulo A, Andrada S, Barajas JL, Fonseca J, Ruiz A, Tribulo R, Tribulo H, Mapletoft RJ, Bó GA (2017). Influence of estrus expression and treatment with GnRH on pregnancy rates in recipients
synchronized with progesterone devices and estradiol and transferred at a fixed time. Reprod Fertil Dev.

[B017] Cedeño A, Tríbulo P, Tríbulo A, Barajas JL, Ortega JA, Andrada JS, Lozano D, Monguillot I, Brandan A, Tribulo R, Tribulo H, Bó GA (2018). Effect of synchronization treatment and estrus expression on conception rates and pregnancy
loses in recipients receiving in vitro produced embryos. Reprod Fertil Dev.

[B018] Davoodi S, Cooke RF, Fernandes AC, Cappellozza BI, Vasconcelos JL, Cerri RL (2016). Expression of estrus modifies the gene expression profile in reproductive tissues on day
19 of gestation in beef cows. *Theriogenology*.

[B019] de la Mata JJ, Bó GA (2012). Estrus synchronization and ovulation using protocols with estradiol benzoate and GnRH
and reduced periods of insertion of a progesterone releasing device in beef heifers. Taurus.

[B020] Frade MC, Frade C, Cordeiro MB, Sá Filho M, Mesquita FS, Nogueira GP, Binelli M, Bertan Membrive CM (2014). Manifestation of estrous behavior and subsequent progesterone concentration at timed-embryo
transfer in cattle are positively associated with pregnancy success of recipients. *Anim Reprod Sci*.

[B021] Garcia-Guerra A, Sala RV, Baez GM, Fosado M, Melo LF, Motta JCL, Leffers L, Walleser EA, Ochoa JC, Moreno JF, Wiltbank MC (2016). Treatment with GnRH on Day 5 reduces pregnancy loss in heifers receiving in vitro-produced
expanded blastocysts. *Reprod Fertil Dev*.

[B022] Hinshaw RH (1999). Formulating ET contracts..

[B023] Jinks EM, Smith MF, Atkins JA, Pohler KG, Perry GA, MacNeil MD, Roberts AJ, Waterman RC, Alexander LJ, Geary TW (2013). Preovulatory estradiol and the establishment and maintenance of pregnancy in suckled
beef cows. *J Anim Sci*.

[B024] Lamb GC, Stevenson JS, Kesler DJ, Garverick HA, Brown DR, Salfen BE (2001). Inclusion of an intravaginal progesterone insert plus GnRH and prostaglandin F2α
for ovulation control in postpartum suckled beef cows. *J Anim Sci*.

[B025] Looney CR, Stutts KJ, Novicke AK, Chiles KC, Tijernia SE, Miranda AR, Romo S, Forrest DW (2010). Advancements in estrus synchronization of Brahman-influenced embryo transfer recipient
females..

[B026] Lopez-Gatius F, Santolaria P, Yaniz JL, Garbayo JM, Hunter RHF (2004). Timing of early foetal loss for single and twin pregnancies in dairy cattle. *Reprod Domest Anim*.

[B027] Madsen CA, Perry GA, Mogck CL, Daly RF, MacNeil MD, Geary TW (2015). Effects of preovulatory estradiol on embryo survival and pregnancy establishment in beef
cows. *Anim Reprod Sci*.

[B028] Mapletoft RJ, Bó GA, International Veterinary Information Service (2016). Bovine embryo transfer. *IVIS Reviews in Veterinary Medicine*.

[B029] Menchaca A, Dutra S, Carrau JM, Sapriza F, Salazar J, de la Mata JJ, Bó GA (2015). Improvement of pregnancy rates by using the 6-day J-Synch protocol in recipient cows transferred
with in vitro produced embryos. Anim Reprod.

[B030] Menchaca A, Dutra S, Carrau JM, Sapriza F, Bó GA (2016). Improvements of the new J-Synch protocol used for fixed-time embryo transfer (FTET) in
recipients transferred with in vitro produced embryos.

[B031] Miles JR, Farin CE, Rodriguez KF, Alexander JE, Farin PW (2005). Effects of embryo culture on angiogenesis and morphometry of bovine placentas during early
gestation. *Biol Reprod*.

[B032] Pereira MHC, Wiltbank MC, Vasconcelos JLM (2016). Expression of estrus improves fertility and decreases pregnancy losses in lactating dairy
cows that receive artificial insemination or embryo transfer. *J Dairy Sci*.

[B033] Perry GA, Smith MF, Roberts AJ, Macneil MD, Geary TW (2004). Effect of ovulatory follicle size on pregnancy rates and fetal mortality in beef heifers. *J Anim Sci*.

[B034] Perry GA, Smith MF, Lucy MC, Green JA, Parks TE, Macneil MD, Roberts AJ, Geary TW (2005). Relationship between follicle size at insemination and pregnancy success. *Proc Natl Acad Sci USA*.

[B035] Perry GA, Smith MF, Roberts AJ, Macneil MD, Geary TW (2007). Relationship between size of ovulatory follicle and pregnancy success in beef heifers. *J Anim Sci*.

[B036] Perry GA, Swanson L, Larimore L, Perry B, Djira GD (2014). Relationship of follicle size and concentrations of estradiol among cows exhibiting or
not exhibiting estrus during a fixed-time AI protocol. *Domest Anim Endocrinol*.

[B037] Perry GA (2017). Efecto de la madurez folicular sobre el establecimiento de la preñez..

[B038] Richardson BN, Hill SL, Stevenson JS, Djira GD, Perry GA (2016). Expression of estrus before fixed-time AI affects conception rates and factors that impact
expression of estrus and the repeatability of expression of estrus in sequential breeding
seasons. *Anim Reprod Sci*.

[B039] Sá Filho MF, Crespilho AM, Santos JEP, Perry GA, Baruselli PS (2010). Ovarian follicle diameter at timed insemination and estrous response influence likelihood
of ovulation and pregnancy after estrous synchronization with progesterone or progestin-based
protocols in suckled *Bos indicus* cows. *Anim Reprod Sci*.

[B040] Sá Filho MF, Santos JEP, Ferreira RM, Sales JNS, Baruselli PS (2011). Importance of estrus on pregnancy per insemination in suckled *Bos indicus*
cows submitted to estradiol/progesterone based timed insemination protocols. *Theriogenology*.

[B041] Sala RV, Carrenho-Sala LC, Fosado M, Tosta LCC, Tosta RD, Stoll M, Moreno JF, Monteiro BM, Baruselli PS, Garcia-Guera A, Wiltbank MC (2016). Comparison of methods for synchronizing recipients of in vitro produced embryos. *Reprod Fertil Dev*.

[B042] Tribulo A, Cedeño AJ, Bernal B, Andrada S, Barajas JL, Ortega J, Oviedo J, Tribulo H, Tribulo R, Mapletoft RJ, Bó GA (2017). Factors affecting pregnancy rates and embryo/fetal losses in recipients receiving in-vitro-produced
embryos by fixed-time embryo transfer. Reprod Fertil Dev.

[B043] Wiltbank MC, Baez GM, Garcia-Guerra A, Toledo MZ, Monteiro PLJ, Melo LF, Ochoa JC, Sartori R (2017). Momento y causas de la pérdida fisiológica de la preñez (no relacionada
con enfermedad) en vacas lecheras lactantes y receptoras de embriones.

